# A pilot study among older adults of the concordance between their self-reports to a health survey and spousal proxy reports on their behalf

**DOI:** 10.1186/s12913-016-1734-6

**Published:** 2016-09-09

**Authors:** Fredric D. Wolinsky, Lioness Ayres, Michael P. Jones, Yiyue Lou, George L. Wehby, Fred A. Ullrich

**Affiliations:** 1 College of Public Health, University of Iowa, Iowa City, Iowa USA; 2 College of Nursing, University of Iowa, Iowa City, Iowa USA

**Keywords:** Bias, Nonresponse, Qualitative research, Survey methods

## Abstract

**Background:**

Proxy respondents are frequently used in health surveys, and the proxy is most often the spouse. Longstanding concerns linger, however, about the validity of using spousal proxies, especially for older adults. The purpose of this pilot study was to evaluate the concordance between self-reports and spousal proxy reports to a standard health survey in a small convenience sample of older married couples.

**Methods:**

We used the *Seniors Together in Aging Research* (*STAR*) volunteer registry at the University of Iowa to identify and consent a cross-sectional, convenience sample of 28 married husband and wife couples. Private, personal interviews with each member of the married couple using a detailed health survey based on the 2012 *Health and Retirement Study* (*HRS*) instrument were conducted using computer assisted personal interviewing software. Within couples, each wife completed the health survey first for herself and then for her husband, and each husband completed the health survey first for himself and then for his wife. The health survey topics included health ratings, health conditions, mobility, instrumental activities of daily living (IADLs), health services use, and preventative services. Percent of agreement and prevalence and bias adjusted kappa statistics (PABAKs) were used to evaluate concordance.

**Results:**

PABAK coefficients indicated moderate to excellent concordance (PABAKs >0.60) for most of the IADL, health condition, hospitalization, surgery, preventative service, and mobility questions, but only slight to fair concordance (PABAKs = −0.21 to 0.60) for health ratings, and physician and dental visits.

**Conclusions:**

These results do not allay longstanding concerns about the validity of routinely using spousal proxies in health surveys to obtain health ratings or the number of physician and dental visits among older adults. Further research is needed in a nationally representative sample of older couples in which each wife completes the health survey first for herself and then for her husband, each husband completes the health survey first for himself and then for his wife, and both spouses’ Medicare claims are linked to their health survey responses to determine not just the concordance between spousal reports, but the concordance of those survey responses to the medical record.

## Background

There are at least three reasons for using proxy interviews in surveys [1]. The first is *reducing selection bias* by using a proxy respondent to gather data on individuals who are unable to respond for themselves due to either a physical or mental health condition. *Increasing efficiency* by reducing data collection costs is the second reason for using proxies, and is achieved by using one knowledgeable proxy respondent to provide information about others residing in the same household. *Improving the accuracy* of the information obtained is the third reason, and is accomplished by using a proxy who is more knowledgeable than the target person about the topics to be covered. Reliance on proxy interviews is common, increases with the age of the target population, and most often involves the spouse [2–4]. For example, 5.1 % of the 12,652 baseline interviews conducted for the (1992) *Health and Retirement Study* (*HRS*) that targeted a nationally representative sample of 51–61 year olds (born in 1931–1941) as well as their spouses or partners regardless of age (mean age = 55.6, range = 23–85) were conducted with proxies, of which 90.1 % were spousal proxies. And of the 8,179 baseline interviews (1993) conducted for the companion *Survey on Assets and Health Dynamics of the Oldest Old* (*AHEAD*) study that targeted a nationally representative sample of those 70 years old and older (born in 1923 or before) as well as their spouses or partners regardless of age (mean age = 76.5, range = 38–103), 10.3 % were conducted with proxies, of which 46.3 % were spousal proxies. Nonetheless, recent reports have questioned the accuracy of proxy interviews [5–8] and much remains unknown about their effects on total survey error.

In previous work, we used data from the *AHEAD* [9, 10] to investigate the accuracy of proxy interviews. Baseline (1993) and biennial follow-up interviews through 2010 were linked to Medicare claims (1991–2010) for 4,910 *AHEAD* participants (19,556 person periods) who were ≥70 years old at baseline. With these data we assessed the relationship between respondent status (self-respondents, self-respondents with the assistance of another person, and proxy-respondents of various types) and the concordance of survey reports with Medicare claims. We found that proxy-respondents were significantly less likely than self-respondents to under-report, but were significantly more likely to over-report on a variety of health conditions, preventative procedures, and health services [11]. After disaggregating proxies, we found that spousal proxies were about as accurate as self-respondents in reporting health services use, but that daughter or son proxies were the most accurate reporters [12]. We hypothesized that their greater accuracy stemmed from their parents’ age-related cognitive decline (as self-respondents or spousal proxies), and found support for this interpretation in the direct relationship between performance on objective cognitive function tests and greater accuracy among self-respondents [13].

Our earlier studies [11–13], however, focused on the concordance between the survey reports and Medicare claims, and thus assessed the accuracy of self vs. proxy respondents compared to administrative data. Those studies were unable to directly examine the agreement between the target person and spousal proxy reports, because the AHEAD does not ask both spouses to report for themselves, and then for their spouses. Furthermore, Medicare claims cannot be used to assess the accuracy of non-billable outcomes such as health ratings, mobility, activity limitations, or dental visits, all of which are key outcomes or covariates in health services research. Because spouses are the most common proxies for older adults [12], we conducted a pilot study in which interviews were conducted with a cross-sectional, convenience sample of 25 married couples. Within each couple, the wife completed the health survey first for herself and then for her husband, and the husband completed the health survey first for himself and then for his wife. We report here on the concordance between self and spousal proxy responses.

## Methods

The Center on Aging (CoA) at the University of Iowa maintains the *STAR* (*Seniors Together in Aging Research*) registry of older adults willing to consider participation in research studies. To access *STAR*, faculty first obtain Institutional Review Board (IRB) approval for their project, including the generic letter describing the proposed study, what would be required of participants, and whether compensation is available. This pilot study was initially approved by the University of Iowa (IRB-01, Protocol Number 201410712) on October 2, 2014 and its most recent continuing review was approved on July 6, 2016. Faculty also specify the characteristics (e.g., age, sex, marital status, and geocodes) that CoA staff use to identify potential participants from the automated *STAR* registry, and then CoA staff mail them the generic study letter. If those individuals are interested in participating, they contact the faculty directly. No information about those who did not choose to participate are provided to the faculty.

We used *STAR* to identify 275 potential participants, and consented (with a waiver of written documentation at the IRB’s request) and interviewed the first 28 male/female couples who made contact, met the eligibility criteria of being ≥65 years old, married, having both spouses willing to participate, residing in Johnson County, Iowa, and consenting to participate. We excluded three couples from these analyses because a glitch in the computer assisted personal interviewing software did not record their spousal reports. In November and December 2014 we interviewed each participant in person in a private room at the University of Iowa with only the participant and the interviewer present.

The survey instrument included major sections from the 2012 *HRS* instrument (available online at http://hrsonline.isr.umich.edu/index.php?p=qnaires). Within couples, each wife completed the health survey first for herself, and following a 10 min break, completed the interview for her husband. Similarly, within couples, each husband completed the health survey first for himself, and following a 10 min break, completed the interview for his wife. The sections of the 2012 *HRS* instrument that we used included health ratings, health conditions, mobility, instrumental activities of daily living (IADLs), health services use, and preventative services. The health ratings were for overall health, vision, hearing, and memory with five response options (excellent, very good, good, fair, or poor), as well as overall health and memory compared to a year ago (about the same, better, or worse). Health conditions (yes or no, as told by a physician) included ever having had arthritis, cancer, high cholesterol, congestive heart failure, diabetes, glaucoma, hip fracture, hypertension, chronic lung disease, osteoporosis, stroke, emotional or nervous or psychiatric conditions, or problems with depression. The mobility items were having difficulties (yes or no) walking several blocks, sitting for 2 h, climbing stairs, stooping or kneeling or crouching, pulling or pushing a heavy object, or lifting a 10-lb bag of groceries. IADLs included having difficulties (yes or no) reading a map, preparing a hot meal, using the telephone, taking medications, and managing money. The health services use items included hospitalization (yes or no, the number of episodes, and the number of nights), outpatient surgeries (yes or no), joint replacement (yes or no), cataract surgery (yes or no), and the number of physician visits (in the past 2 years and in the past year) and dentist visits (in the past 2 years). Preventative services (yes or no) were having had flu, pneumonia, and shingles vaccinations, bone density testing, colon cancer screening, and mammography (for women) or prostate specific antigen (PSA) testing (for men).

We first describe the sample of husbands and wives in terms of their demographic, socioeconomic, health, and cognitive characteristics. Then, we present the percent agreement between self-reports and spousal proxy reports, the percent of spousal proxies who under-reported (based on the spouse’s self-reports) or over-reported for their spouses, and prevalence and bias adjusted kappa (PABAK; either binary or ordinal) coefficients for concordance. Unlike kappa which only adjusts for chance, PABAKs also adjust for differences in the marginal rates of disease prevalence between the self and proxy responses, as well as for prevalence rates that deviate from a 50/50 split [14]. PABAKs may be categorized as follows: < 0.00 = poor agreement between raters; 0.00–0.20 = slight agreement between raters; 0.21–0.40 = fair agreement between raters; 0.41–0.60 = moderate agreement between raters; 0.61–0.80 = substantial agreement between raters; and > 0.81 = excellent agreement between raters [15]. Based on the literature [2, 11], we expected concordance would be greatest for outcomes that were more salient, involved invasive treatments, were relatively rare, or required constant monitoring coupled with potentially invasive treatment. In contrast, we expected rather frequent and less salient outcomes would have the lowest concordance. Finally, based on our prior work [11–13], we explored whether the concordance between self and spousal proxy reports differed based on the sex of the spousal proxy or the cognitive ability of the self-respondent using chi-square tests.

## Results

Based on the target person responses, mean age was 75 years old (range = 64–93, median = 74.5), mean current marriage length was 46 years (range = 4–68, median = 48), 72 % had completed college, with 58 % having some postgraduate training. Seventy percent rated their health as either excellent or very good with 78 % saying their health was about the same as last year and 14 % indicating that their health had improved. About half (54 %) rated their memory as either excellent or very good with 82 % saying it was about the same as last year and 16 % indicating that it had declined. The most common health conditions were arthritis (64 %), hypertension (56 %), and having had an emotional, nervous, or psychiatric condition (26 %). One quarter (26 %) had been hospitalized in the past two years, and the mean number of physician visits in that period was 8.0 (range = 1–30, median = 7.0) with 4.6 (range = 0–24, median = 3.5) of those visits occurring in the last year. Finally, on a modified version of the Telephone Interview for Cognitive Status (TICS; including the 10-word immediate and delayed recall tests, serial 7 s, and mental status items; range = 0–35), the mean score was 25.7 (median = 26.0, range = 13 to 33), which is noticeably better than national norms [16].

Table 1 contains the percent of agreement and PABAK coefficients for the health ratings. Agreement was best for health (70.0 %) and memory (75.5 %) when compared to a year ago with PABAKs indicating moderate or substantial concordance. Agreement was quite low (≤56.0 %) for the current health ratings, however, with PABAKs indicating only slight to fair concordance, except for overall health which had moderate concordance. Spousal proxies tended to rate the target person’s overall health and hearing lower, but their memory higher. As shown in Table 2, agreement on the health conditions was much better, ranging from 70.8 to 98.0 %, with the PABAK coefficients indicating moderate concordance for high cholesterol and emotional, nervous, or psychiatric problems; substantial concordance for arthritis, hypertension, osteoporosis, and depression; and, excellent concordance for all of the other health conditions.

**Table 1:** Percent Agreement and PABAKs for 6 Functional Health Ratings between Self and Spousal Proxy Responses

Survey items	Percent agreement between self and proxy responses	Percent of proxy responses indicating worse health	Percent of proxy responses indicating better health	Percent of discrepancies ≥2 response rating categories	PABAKs
How would you rate your …; would you say it was excellent, very good, good, fair, or poor?					
Health	56.0 %	32.0 %	12.0 %	6.0 %	0.45
Vision	27.1 %	35.4 %	37.5 %	16.6 %	0.09
Hearing	36.4 %	40.8 %	22.4 %	16.3 %	0.21
Memory	32.7 %	22.4 %	44.9 %	8.2 %	0.18
Compared to 1 year ago, how would you rate your …; would you say that it is better now, about the same, or worse?					
Health	70.0 %	18.0 %	12.0 %		0.55
Memory	75.5 %	12.2 %	12.2 %		0.63

**Table 2:** Percent Agreement and PABAKs for 13 Health Conditions between Self and Spousal Proxy Responses

Survey items	Percent agreement between self and spousal proxies	Percent of spousal proxies not indicating the condition when the self did	Percent of spousal proxies indicating the condition when the self did not	PABAKs
Has a doctor ever told you that you have …?				
Arthritis	86.7 %	11.1 %	2.2 %	0.73
Cancer (other than skin)	93.9 %	2.0 %	4.1 %	0.88
High Cholesterol	70.8 %	18.8 %	10.4 %	0.42
Congestive Heart Failure	98.0 %	2.0 %	0.0 %	0.96
Diabetes	93.9 %	4.1 %	2.0 %	0.88
Glaucoma (treated by doctor)	93.6 %	4.3 %	2.1 %	0.87
Hip Fracture	98.0 %	2.0 %	0.0 %	0.96
Hypertension	83.3 %	12.5 %	4.2 %	0.67
Chronic Lung Disease (emphysema or bronchitis)	98.0 %	0.0 %	2.0 %	0.96
Osteoporosis	84.8 %	8.7 %	6.5 %	0.70
Stroke	98.0 %	2.0 %	0.0 %	0.96
Emotional, Nervous, or Psychiatric Problems	79.6 %	16.3 %	4.1 %	0.59
Problems with Depression	87.5 %	6.3 %	6.3 %	0.75

Table 3 contains the percent of agreement and PABAK coefficients for the mobility and IADL items. The percent of agreement on mobility was good (63.3 to 85.7 %), with the PABAK coefficients indicating fair concordance for stooping or crouching or kneeling, moderate concordance for sitting and climbing stairs, substantial concordance for pulling or pushing large objects or lifting groceries, and excellent concordance for walking. Notably, spousal proxies under-reported the target persons’ difficulties with walking, sitting, and stooping or crouching or kneeling. Agreement on difficulties with the IADL items was very good (85.7 to 95.9 %), with the PABAK coefficients indicating substantial concordance for using a map and taking medications, and excellent concordance for preparing hot meals, using the telephone, and managing money.

**Table 3:** Percent Agreement and PABAKs for 6 Mobility and 5 IADL Questions between Self and Spousal Proxy Responses

Survey items	Percent agreement between self and spousal proxies	Percent of spousal proxies not indicating difficulty when the self did	Percent of spousal proxies indicating the difficulty when the self did not	PABAKs
Mobility				
Because of a health problem, do you have any difficulty with … ?				
walking several blocks	73.5 %	18.4 %	8.2 %	0.84
sitting for about 2 h	79.6 %	20.4 %	0.0 %	0.59
climbing several flights of stairs without resting	73.5 %	6.1 %	20.4 %	0.47
stooping, kneeling, or crouching	63.3 %	24.5 %	12.2 %	0.27
pulling or pushing large objects like a living room chair	81.3 %	8.3 %	10.4 %	0.63
lifting or carrying weights over 10 lb, like a bag of groceries	85.7 %	8.2 %	6.1 %	0.71
Instrumental IADLs				
Because of a health or memory problem, do you have any difficulty with … ?				
using a map to figure out how to get around in a strange place	85.7 %	2.0 %	12.2 %	0.71
preparing a hot meal	91.8 %	2.0 %	6.1 %	0.84
making phone calls	91.8 %	0.0 %	8.2 %	0.84
taking medications	87.8 %	6.1 %	6.1 %	0.76
managing your money, such as paying your bills and keeping track of expenses	95.9 %	0.0 %	4.1 %	0.92

Table 4 contains similar data for the health services. For the more salient services (hospitalization, and outpatient, joint, or cataract surgery) the percent of agreement is very good (74.5 to 100 %), with the PABAK coefficients indicating moderate (hospitalization, outpatient surgery), substantial (number of hospital episodes and nights), or excellent (joint replacement and cataract surgery) concordance. For the number of physician visits, however, the percent agreement (and PABAK coefficients) were low (slight) for both the 1- and 2-year windows, although the percent agreement improved when it was loosely defined as being within +/- two visits. The percent agreement (and PABAK coefficient) for the number of dental visits was also low (slight), although the percent agreement also improved when it was loosely defined as being within +/- two visits. The amounts of under- and over-reporting by the spousal proxies was reasonably balanced, except for outpatient surgery which was more likely to be under-reported and dentist visits which were more likely to be over-reported.

**Table 4:** Percent Agreement and PABAKs for 9 Health Services between Self and Spousal Proxy Responses

Survey items	Percent agreement between self and spousal proxies	Percent of spousal proxies not indicating service use when the self did (or under-reporting)	Percent of spousal proxies indicating service use when the self did not (or over-reporting)	PABAKs
Hospitalization				
In the last 2 years, have you been a patient in the hospital overnight?	85.4 %	10.4 %	4.2 %	0.58
How many different times were you a patient in the hospital in the last 2 years?	78.0 %	8.0 %	14.0 %	0.75
How many different nights were you a patient in the hospital in the last 2 years?	76.0 %	10.0 %	14.0 %	0.73
Outpatient Surgery				
Not counting overnight hospital stays, in the last 2 years have you had outpatient surgery?	74.5 %	19.1 %	6.4 %	0.42
Joint Replacement Surgery				
Have you ever had joint replacement surgery?	98.0 %	0.0 %	2.0 %	0.94
Cataract Surgery				
Have you ever had cataract surgery?	100 %	0.0 %	0.0 %	1.00
Physician Visits				
Aside from hospital stays or outpatient surgery, in the past 2 years how many times have you talked to a medical doctor about your health, including emergency room, clinic visits, or house calls?	14.9 %	46.8 %	38.3 %	0.20^a^
Allowing for +/−2 visits to be agreement	57.5 %	25.5 %	17.0 %	N/A
How many of those visits were in the last year?	6.8 %	43.2 %	50.0 %	0.17^a^
Allowing for +/−2 visits to be agreement	47.7 %	25.0 %	27.3 %	N/A
Dentist Visits				
How many times have you seen a dentist in the last 2 years?	22.0 %	22.0 %	54.0 %	0.15
Allowing for +/−2 visits to be agreement	72.0 %	4.0 %	24.0 %	N/A

^a^ = truncated at >8 visits

*N/A* PABAKs cannot be calculated for +/−2 visits

Table 5 contains the percent of agreement and PABAK coefficients for the preventative service items. With the exception of pneumonia vaccinations, the percent of agreement was high (78.3 to 93.8 %), with the PABAK for colon cancer screening indicating moderate concordance, the PABAKs for shingle vaccinations and bone density testing indicating substantial concordance, and the PABAKs for flu shots, mammograms, and PSA testing indicating excellent concordance. Spousal proxies were more likely to under-report pneumonia vaccinations and bone density testing.

**Table 5:** Percent Agreement and PABAKs for 6 Preventative Services between Self and Spousal Proxy Responses

Survey items	Percent agreement between self and spousal proxies	Percent of spousal proxies not indicating use of the preventative service when the self did	Percent of spousal proxies indicating use of the preventative service when the self did not	PABAKs
In the last … years, have you had a …?				
Flu Vaccination (last 2 years)	93.8 %	4.2 %	2.1 %	0.88
Pneumonia Vaccination (last 5 years)	58.1 %	25.8 %	16.1 %	0.16
Shingles Vaccination (ever)	87.5 %	7.5 %	5.0 %	0.75
Bone Density or Bone Scan Test (ever)	81.8 %	15.9 %	2.3 %	0.64
Colonoscopy, Sigmoidoscopy, or Other Screening for Colon Cancer (last 4 years)	78.3 %	10.9 %	10.9 %	0.57
Mammogram or X-Ray of the Breast (women only; last 2 years)	91.3 %	4.3 %	4.3 %	0.83
PSA Blood Test (men only; ever)	90.9 %	0.0 %	9.1 %	0.82

Finally, the analyses exploring the influence of the sex of the spousal proxy and the cognitive ability of the self-respondent on the agreement between the self and spousal proxy responses to the 40 binary outcomes in Tables 1, 2, 3, 4 and 5 was uninformative, perhaps due to the reduced sample size. In terms of the sex of the spousal proxy, p values ≤0.10 were observed for only four outcomes (high cholesterol, osteoporosis, bone density testing, and shingles vaccinations), which is exactly the number that would be expected by chance (data not shown). In all four cases, however, female spousal proxies were less likely to agree with their husband’s self-reports. In terms of the cognitive ability (classified in tertiles on the TICS overall score) of the self-respondent, *p* values ≤0.10 were observed for only two outcomes (vision and outpatient surgery), which is but half of the number that would be expected by chance (data not shown). In both cases, the better the cognitive ability of the self-respondent, the greater the agreement with their spouse’s report.

## Discussion

In a pilot study using a cross-sectional, convenience sample of 25 married male/female couples, we conducted personal interviews to evaluate the concordance between self and spousal proxy reports. Within couples, each wife completed the health survey first for herself, and following a 10 min break, then completed the interview for her husband. Similarly, within couples, each husband completed the health survey first for himself, and following a 10 min break, then completed the interview for his wife. The interviews included the health ratings, health conditions, mobility, IADLs, health services use, and preventative services sections of the 2012 *HRS* instrument.

This approach allowed us to go beyond assessing accuracy simply by comparing survey reports from different respondent status categories (self-respondents, self-respondents with the assistance of another person, and proxy-respondents of various types) with administrative claims data [11–13]. In particular, it allowed us to assess the actual agreement between self and spousal proxy responses, and to include in those assessments questions that cannot be compared to Medicare claims (health ratings, mobility, IADLs, dental visits). This is important because (a) the use of proxies in health surveys is common, (b) the most widely used proxy in the *HRS*, *AHEAD*, and other health surveys is a spousal proxy, and (c) the health ratings, mobility, IADL, and dental visit items are commonly used in health services research but are not available in administrative claims data. We expected agreement between self and spousal proxy responses to be highest for the more salient conditions, and for those that involved invasive treatments or were relatively rare, while agreement would be lowest for more frequent and less salient outcomes and the health ratings.

Because our sample was drawn from the volunteer registry (*STAR*) at a research intensive university in a small city, the majority of participants were white, long-time married, highly educated, and had good health and cognitive function. Thus, sample is clearly neither nationally representative nor representative of couples of all ages, race and ethnic groups, or marital statuses. That said, given these characteristics of our convenience sample, we expected high levels of concordance between self and spousal proxy responses based on previous reports linking one or more of these characteristics with concordance rates [1–8, 11–13]. For the most part that is what we found. Not surprisingly [2, 11], we found PABAK coefficients indicating moderate to excellent concordance between self and spousal proxy responses for the health conditions, hospitalizations, and surgeries. The highest and most consistent levels of concordance (substantial to excellent), however, were observed for the IADLs. PABAK coefficients for the mobility and preventative services questions also indicated moderate to excellent concordance, except for pneumonia vaccinations which were poor. Finally, agreement between self and spousal proxy responses for the current year health ratings and physician and dental visit questions were the worst, ranging only from slight to fair concordance.

While our results are informative, further research on the use of proxies in health surveys is needed. Specifically, research is needed that uses a nationally representative sample of couples from all marital statuses in which for example, each wife completes the health survey first for herself and then for her husband, each husband completes the health survey first for himself and then for his wife, and both spouses’ medical records are linked to their health survey responses. Such a study would be easiest to conduct using older couples whose members are participants is Medicare Part A and B so that their administrative claims data would be available. In addition, performance-based IADL and mobility testing is needed at the conclusion of the self-reported interviews to establish the concordance on these items because they are not available in the Medicare claims. Only a rich data set like this would allow for both the assessment of concordance between spousal reports, and the concordance of those survey responses to the medical record and IADL and mobility performance test results.

## Conclusion

The agreement and concordance levels for the health ratings, physician and dental visits questions are disappointing. Furthermore, given the characteristics of the husbands and wives in our convenience sample, these findings likely underestimate the magnitude of the problem nationally. And that has potentially important implications because the most common proxies used in the *HRS* and other health surveys of older adults are spouses, and these data suggest substantial disagreements between husbands’ and wives’ responses for themselves compared to their spouses’ proxy responses on their behalf. Moreover, in health services research the health ratings are commonly used as key covariates, and the number of physician and dental visits are commonly used as key outcomes. Therefore, researchers using spousal proxies should acknowledge this limitation and examine differences in results across factors that can affect concordance such as age, education, or cognitive status.

## Abbreviations

AHEAD: Survey on assets and health dynamics among the oldest old
CoA: Center on aging
HRS: Health and retirement study
IADLs: Instrumental activities of daily living
IRB: Institutional Review Board
PABAK: Prevalence and bias adjusted kappa
PCORI: Patient-Centered Outcomes Research Institute
PSA: Prostate specific antigen
STAR: Seniors Together in Aging Research
TICS: Telephone interview for cognitive status
